# Realism and robustness require increased sample size when studying both sexes

**DOI:** 10.1371/journal.pbio.3002456

**Published:** 2024-04-11

**Authors:** Szymon M. Drobniak, Malgorzata Lagisz, Yefeng Yang, Shinichi Nakagawa

**Affiliations:** 1 Evolution & Ecology Research Centre and School of Biological, Earth and Environmental Sciences, University of New South Wales, Sydney, Australia; 2 Institute of Environmental Sciences, Jagiellonian University, Kraków, Poland; 3 Theoretical Sciences Visiting Program, Okinawa Institute of Science and Technology Graduate University, Onna, Japan

## Abstract

A recent article claimed that researchers need not increase the overall sample size for a study that includes both sexes. This Formal Comment points out that that study assumed two sexes to have the same variance, and explains why this is a unrealistic assumption.

Female subjects have been historically excluded from biomedicine and other related areas of study [1]. Such exclusion has disadvantaged females and prevented a fuller understanding of biology. Therefore, in 2016, the National Institute of Health (NIH) mandated that all NIH-funded animal and human studies consider sex as a biological variable (e.g., [2]). Yet, sex as a biological variable has not been welcomed with open arms, most likely because many researchers believe they need to increase the overall sample size with 2 sexes compared to using only 1 sex (e.g., [2]). Recently, Philips and colleagues published a *PLoS Biology* article titled “*Statistical simulations show that scientists need not increase overall sample size by default when including both sexes in in vivo studies*” [3]. As indicated in their title, the authors have concluded and recommended no increase in sample size with both sexes, which was based on a set of simulations exploring a simple but—as they claim—likely biological scenario. Their conclusion is great news for researchers who feared coping with increased experiment sizes and costs.

However, Philips and colleagues have assumed homoscedasticity between the 2 sexes, meaning variances or standard deviations of the sexes are the same throughout their simulations. Here, we first explain why such an assumption is biologically unrealistic and why heteroscedasticity between 2 sexes should be the norm rather than the exception by pointing out a wealth of empirical evidence and evolutionary arguments. We then show the results from a simulation study expanding Philips and colleagues’ work by incorporating heteroscedasticity. Our results clearly indicate that we need to increase the overall sample size to have robust statistical inference. Further, we provide statistical recommendations to deal with heteroscedasticity. We also briefly touch on what funding agencies and ethics committees can do, given our results.

## Why do 2 sexes have different variances?

**Fig 1:**
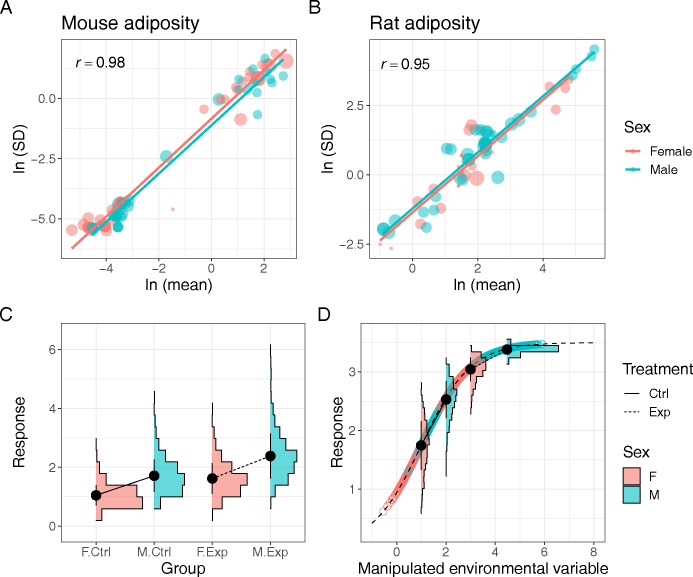
**(A, B) An example of a strong mean and variance relationship or Taylor’s law.** The offspring adiposity data is taken from a meta-analysis of 12 studies on the transgenerational effects of obesogenic diets in mice and rats [5]. Data for female and male offspring is plotted separately, correlations are calculated for both sexes, point size represents sample sizes. **(C, D) Heteroscedasticity resulting from the properties of data-generating processes.** Heteroscedastic variances may be associated with varying means if underlying distribution exhibits a mean–variance relationship (C, here a log-normal example), or if ceiling/floor effects result from nonlinear functions mapping treatment/sex effects into the phenotypic space (D, here assuming logistic mapping). See Supplementary Information for details (the data underlying this figure can be found in https://doi.org/10.5281/zenodo.10205440).

There are 2 major reasons why males and females have different variances in their traits and responses. The one is due to Taylor’s law, where an increase in variance accompanies an increase in a mean. The law was first used to describe organismal aggregation patterns, but this mean–variance relationship seems to be ubiquitous [4]. Often correlations are over 0.9 between mean and variance (standard deviation) on the logarithm scale, as shown in an example data from a meta-analysis on rodent diet manipulations [5] (Fig 1A–1C). Taylor’s law means that when there are sex differences in mean, there are also unavoidable differences in variances (i.e., heteroscedasticity). In their simulation, Philips and colleagues showed that a sex difference in treatment effects would usually increase statistical power. Yet, heteroscedasticity reduces power (see the next section). Given the empirically observed widespread mean–variance relationship, biologists often use CV (coefficient of variation; a mean standardised standard deviation) to compare variability among traits and responses. The other reason is an evolutionary inevitability, where 2 sexes have been subject to different natural and sexual selection forces. Indeed, the evolutionary and biomedical literature comparing CVs has found clear and widespread differences between sexes [6,7]. Of relevance, we have demonstrated this very point in mice [8,9]. Therefore, even if there are no sex differences in mean, we should expect heteroscedasticity between the 2 sexes.

## Building upon the simulation study

**Fig 2:**
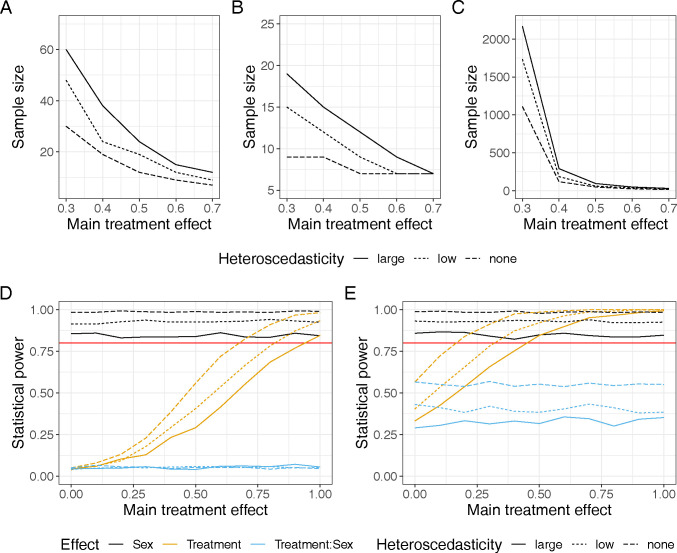
Simulation results. Top row: sample sizes required to achieve power of at least 80% under 3 heteroscedasticity scenarios (line type coding) and for varying magnitudes of treatment effect (x axis, limited to moderate-size effects); (A)—no sex-by-treatment interaction, (B)—interaction present, stronger treatment effect in more variables sex, (C)—same, but stronger treatment effect in less variable sex. Bottom row: power to estimate model effects (colour-coded) under 3 heteroscedasticity scenarios (line type) without (D) and with (E) sex-by-treatment interaction, with varying treatment effect size magnitude (*x* axis); the simulated interaction modified the magnitude of sex effect between treatment groups, but not its direction. Solid red line—power threshold of 80% (the data underlying this figure can be found in https://doi.org/10.5281/zenodo.10205440).

As mentioned above, we conducted a simulation study in which we added, to Philips and colleagues’ simulation, 2 more scenarios with “small” and “large” heteroscedasticity, where one sex had approximately 44% and 73% larger standard deviation than the other (50% and 100% increase in sex-specific variance, respectively). As shown in Fig 2A and 2B, statistical power is lower, sometimes substantially so, when heteroscedasticity exists. This pattern is consistently true regardless of whether there is an interaction between sex and treatment. Furthermore, we investigated to see how much larger sample sizes are required when 2 sexes are heteroscedastic. To achieve the nominal 80% power, the large heteroscedasticity scenario required twice as many sample sizes for a range of effect sizes, regardless of the interaction (Fig 2C–2E). This is especially true when the effect size (*d* or standardised mean difference) is less than 0.5, which would be common in non-pharmacological/toxicological studies. Importantly, the increased sample size requirements are not alleviated by using methods designed to account for heteroscedastic variance (for explanations of such methods and additional simulations using the methods, see the next section).

We note that our scenarios may represent the more extreme heteroscedasticity cases, but that they are not by any means rare or exceptional. Nevertheless, to test the robustness of our finding, we added an extra condition, constraining average variance between the sexes to remain approximately constant, and we found that such condition also leads to often larger sample size requirements in sex-heteroscedastic scenarios. Therefore, there is a clear need for larger sample sizes in a study measuring heteroscedastic traits and responses, especially when 2 sexes have different average responses. Taken together, we arrive at 2 conclusions under biological realistic scenarios (at least under the scenarios we simulated) or, i.e., with the presence of heteroscedasticity: (1) if one assumes homoscedasticity like Philips and colleagues, one’s study is likely to be underpowered; and (2) if one does not model or deal with heteroscedasticity in analysis, it may lead to inflated Type I error rates exceeding 5%. All relevant R code and simulation results are found in the supplementary code document available via the webpage (see Supplementary Information, webpage: https://szymekdr.github.io/230713_power_sex_sim/).

## Empirical and analytical recommendations

Therefore, we suggest researchers consider increasing sample size when including both sexes in their studies, unlike what Philips and colleagues recommended. In earlier work, we recommend that a study consider increasing the sample size for the sex with higher variability, as this is more efficient in gaining more power than increasing sample sizes in a balanced manner [8]. Also, there are 2 ways to deal with data with heteroscedasticity [10]. First, we can use heteroscedasticity-consistent standard error (variance) estimators, often known as “sandwich estimators,” with which standard errors are calculated correctly, usually increasing standard error or confidence intervals, maintaining the nominal Type I error rate (note that in many statistical software and packages, sandwich estimators are available and there are many types of sandwich estimators, e.g., [11]). Second, we can quantify homoscedasticity explicitly, using a generalized least squares model (for more details, see [10]; alternatively, one can use location-scale models; for example, [12]), and—with sufficient replication across grouping levels—decompose it into its causal constituents. Such explicit modelling is our preferred option because differences in variances can tell biological and evolutionary stories, especially when trends in variability are opposite of what is expected from Taylor’s law (e.g., ceiling and floor effects can create such patterns [13]; Fig 1D).

Explicit modelling of heteroscedasticity also aligns with ongoing efforts to make biomedical science more aware of individual differences (e.g., precision or personalised medicine). Adhering to adequate sample size requirements and including both sexes in empirical research by default, will improve the generalisability of research and encourage more biologically realistic planning of future experiments. However, meaningful modelling of heteroscedasticity would still require large sample sizes for both sexes. Of relevance, sample sizes, required in the generalised least-squares framework with variance heteroscedasticity modelled explicitly, yield comparable sample size requirements to scenarios not modelling heteroscedasticity. Sandwich estimators equalise sample size requirements for low and large heteroscedasticity scenarios, but simple size requirements remain markedly higher than in homoscedastic scenarios (see Supplementary Information, webpage: https://szymekdr.github.io/230713_power_sex_sim/ Section 8).

## Concluding remarks

Our aim of this commentary is not to highlight difficulties in including sex as a biological variable in a study, discouraging sex inclusion. Quite the opposite, we believe sex inclusion is a critical element of study design for generalisability, agreeing with Philips and colleagues. However, sex inclusion is not a free ride, unlike what Philips and colleagues suggested. We urge that funding bodies and ethics committees recognise that if they ask for sex inclusion, they will need to allow researchers to have more subjects and budget to gain robust statistical inference and, therefore, robust conclusions (cf. [14]). There is no way around this biological and statistical reality.

**Supplementary Information:** An HTML file containing 8 sections: Sections 1–3, different simulation scenarios; Section 4, the impact of sample size; Section 5, simulating sample size; Section 6, source of heterogeneity, Section 7, Taylor’s law; and Section 8, additional simulation scenarios (webpage: https://szymekdr.github.io/230713_power_sex_sim/ or underlying files and data can be found in: https://doi.org/10.5281/zenodo.10205440).
